# Ailanthone blocks mitophagy to promote mtDNA leakage through BAX-BAK1 pores and suppress hepatocellular carcinoma cell proliferation

**DOI:** 10.3389/fphar.2024.1509482

**Published:** 2024-12-11

**Authors:** Yan Qin, Ying Gao, Dan Wu, Qing-Qing Liu, Chang Su, Guan Liu, Le Yang, Ming-Gao Zhao, Jing-Yue Yao

**Affiliations:** Precision Pharmacy and Drug Development Center, Department of Pharmacy, Tangdu Hospital, Air Force Medical University, Xi’an, Shaanxi, China

**Keywords:** hepatocellular carcinoma, ailanthone, PINK1-PRKN, mtDNA, BAX-BAK1, inflammation

## Abstract

**Introduction:**

Hepatocellular carcinoma (HCC), the third leading cancer mortality worldwide, shows rising incidence. The mitochondria in HCC cells are prone to damage from metabolic stress and oxidative stress, necessitating heightened mitophagy for mitochondrial homeostasis and cell survival. Thus, mitophagy inhibition is a promising HCC therapy. The traditional Chinese medicinal herb ailanthone have proved promote mitochondrial dysfunction and inhibits HCC. However, the underlying mechanism remains unclear.

**Methods:**

CCK8 assay was applied to detect the proliferation. JC-1, MitoTracker Red/Green and MitoSOX staining were applied to detect the mitochondrial homeostasis. Inflammatory factors were analysed via ELISA and WB assay. Mitochondria and cytoplasm separation, genome extraction and qPCR were used to detect mitochondrial DNA (mtDNA) leakage. Mitochondria ultrastructure was detected by transmission electron microscopy. WB and IHC experiments were applied to detect protein expression. Protein-protein interactions detected by immunoprecipitation and immunofluorescence imaging. The *in vivo* antitumor effect was validated by the xenograft mouse model.

**Results:**

In this study, we demonstrated the potent anti-HCC properties of ailanthone and revealed its molecular mechanism. *In vitro* studies demonstrated that ailanthone effectively inhibited PINK1-PRKN mediated mitophagy and promoted BAX-BAK1 mitochondrial pores formation through PRKN inhibition. This process led to the mitochondrial mtDNA leakage into the cytoplasm, which subsequently triggered the induction of inflammatory factors. The inhibition of mitophagy and the activation of inflammatory response ultimately led to HCC proliferation inhibition. In vivo studies demonstrated that ailanthone exhibited stronger anti-HCC activity than 5-Fluorouracil (5-FU), with no significant adverse effects on animal body weight or the physiological functions of vital organs.

**Conclusion:**

This study highlighted the efficacy of ailanthone against HCC and elucidated its underlying molecular mechanisms, suggesting the promising therapeutic potential of ailanthone for HCC.

## 1 Introduction

Hepatocellular carcinoma (HCC) has shown a steadily increasing mortality rate over the past decades, with a 5-year relative survival rate of only 21% (Siegel et al., 2023). Currently, tyrosine kinase inhibitors (TKIs), such as sorafenib and lenvatinib, stand as the first-line treatment for patients with advanced HCC. However, TKIs are associated with severe side effects and drug resistance (Kudo et al., 2018; Tang et al., 2020). Therefore, novel anti-HCC therapeutic agents are urgently needed.

Mitochondria serve as the primary drivers of adenosine triphosphate (ATP) production in biological processes and play a significant role in human cancer pathogenesis. Hepatocytes are abundant in mitochondria, which constitute approximately 13%–20% of liver volume (Ma et al., 2020). Thus, mitochondrial status is especially important for the development of HCC. Recently, a growing consensus has suggested that disrupting mitochondrial homeostasis could be a promising therapeutic strategy for HCC (Yao J. et al., 2022; Feng et al., 2023; Zhang et al., 2024). Mitophagy, a form of selective macroautophagy, is critical for maintaining a functional network of tumour mitochondria via the autophagosome-lysosome system, primarily targeting and removing damaged or dysfunctional mitochondria (Rugarli and Langer, 2012). The process of mitophagy is often manipulated by cancer cells as a mechanism for sustaining their metabolic reprogramming and unrestrained growth (Zong et al., 2016). In recent years, mitophagy, which is responsible for regulating mitochondrial homeostasis, also has attracted an increasing attention in tumour drug resistance (Zhang et al., 2023; Wang et al., 2024). Most chemotherapeutic drugs induce cytotoxic effects by inducing mitochondrial dysfunction and oxidative stress. However, mitophagy facilitates cell survival by eliminating damaged mitochondria to better adapt to aggressive conditions (Panigrahi et al., 2020). Additionally, mitophagy inactivation also proved to accompany full-blown inflammatory responses driven by unrecoverable mitochondrial dysfunction (Marchi et al., 2023). Mitochondrial dysfunction can release mitochondrial damage-associated molecular patterns (mtDAMPs), such as ATP and mitochondrial DNA (mtDNA), triggering inflammation via the NLRP3 inflammasome and/or the cGAS-STING pathway, subsequently activating innate immunity (Nakahira et al., 2011; White et al., 2014; West et al., 2015). Activation of the immune system in tumours upon treatment is the cornerstone of current immunotherapies (Sharma and Allison, 2015). Therefore, inhibiting mitophagy and aggravating the inflammatory response may hold promise as a promising therapeutic strategy for HCC.

Recent insights into mitophagy suggest that PTEN-induced putative kinase 1 (PINK1) and parkin RBR E3 ubiquitin protein ligase (PRKN) play central roles in mitochondrial quality control. PINK1 is a serine/threonine kinase that phosphorylates PRKN and activates its E3 ubiquitin ligase activity. Then, PRKN ubiquitylates outer mitochondrial membrane proteins, which in turn recruit other proteins to mitochondria to initiate mitophagy (Pickrell and Youle, 2015). Notably, several Chinese herbal extracts, such as oroxylin A and alantolactone, exert antitumoural effects by inhibiting PINK1-PRKN-mediated mitophagy (Kang et al., 2019; Yao J. Y. et al., 2022). Mitochondrial dysfunction leads to the leakage of mtDNA from BCL-2-associated X (BAX)-BCL-2 antagonist/killer 1 (BAK1) proapoptotic pores in the outer mitochondrial membrane, triggering inflammation (Marchi et al., 2023). Loss of PINK1 or PRKN activates cGAS-STING signalling, most likely via mtDNA leakage from defective mitochondria that are not cleared by mitophagy, leading to an inflammatory phenotype (Sliter et al., 2018; Bock and Tait, 2020). These findings indicate that suppressing PINK1-PRKN pathway-mediated mitophagy is an ideal approach to combating HCC.

Ailanthone, a primary quassinoid derived from the traditional Chinese medicinal herb *Ailanthus altissima*, exhibits strong antitumour activity *in vitro* and *in vivo* (Zhuo et al., 2015; Yu R. et al., 2022). Previous studies revealed that ailanthone triggers HCC cells apoptosis by inducing mitochondrial dysfunction (Zhuo et al., 2015). Further research is needed to investigate the specific molecular events of mitochondrial dysfunction caused by ailanthone. In the present study, we revealed that ailanthone blocks mitophagy through inhibiting the PINK1-PRKN pathway, leading to failed mitochondrial renewal and disruption of mitochondrial homeostasis. In addition, we further investigated the effects of ailanthone on mitophagy and its correlation with inflammation to provide insights into the underlying mechanism of its anti-HCC effects.

## 2 Materials and methods

### 2.1 Reagents

Ailanthone (C_20_H_24_O_7_, MW: 376.4, purity: 99.78%, TQ0209) and carbonyl cyanide 3-chlorophenylhydrazone (CCCP, C_9_H_5_ClN_4_, MW: 204.6, purity: 99.64%, T7081) were purchased from TargetMol, USA., was dissolved in dimethyl sulfoxide (DMSO; Sigma-Aldrich, V900090), made into a 0.2 M stock solution and stored at −20°C. MSN-50 was purchased from MedChemExpress (C_36_H_38_BrN_3_O_6_, MW 688.61; purity 98.68%; HY-118948), dissolved in DMSO and prepared as a 0.1 M stock solution and stored at −20 °C. For animal studies, ailanthone dissolved in physiological saline, the final concentration was 0.2 mg/mL and stored at 4°C. A stock solution of 5-Fluorouracil (5-FU) (25 mg/mL; Southwest Pharmaceutical Co., Ltd., China, NO. 20220527) was prepared in physiological saline and stored at 4°C.

### 2.2 Cell culture

The HCC cell lines MHCC-97H, HCC-LM3, HepG2 and Huh7 cells were obtained from Cell Bank of the Chinese Academy of Sciences (Shanghai, China). All the cells were maintained in Dulbecco’s Modified Eagle Medium (DMEM; Thermo Fisher Scientific, Gibco™, 12100061), supplemented with 10% fetal bovine serum (Wisent Bio Products, 080–910), Penicillin-Streptomycin (×100, containing 10,000 units/mL of penicillin and 10,000 μg/mL of streptomycin; Thermo Fisher Scientific, Gibco™, 15140148) and cultured in a humidified environment with 5% CO_2_ at 37°C.

### 2.3 Cell proliferation assay

10% CCK8 reagent (MedChemExpress, HY-K0301) was added in ailanthone treated cells for 1 h. The absorbance was measured at 450 nm by Universal Microplate Reader (EL800, BIO-TEK Instruments Inc., Winooski, VT, United States).

### 2.4 Detection of mitochondrial membrane potential (MMP)

MMP was detected with the JC-1 stain according to the manufacturer’s protocol (Beyotime Biotechnology, C2006). For the determination of MMP by flow cytometry, cells were gently collected and detected in accordance with the instructions of the Becton-Dickinson FACS Calibur flow cytometry instrument. For JC-1 imaging, we stained JC-1 stain in accordance with the instructions and acquired images using Cell Imaging Multi-Mode Reader (BioTek Cytarion1) driven by Gen5 software.

### 2.5 Mitochondria and cytoplasm protein extraction and detection

Mitochondrial and cytoplasmic protein extraction from Huh7 cells was performed with a Mitochondria Isolation Kit for Cultured Cells (Thermo, 89874) according to manufacturer’s instructions. 2×10^7^ cells were obtained by centrifuging the harvested cell suspension at 850 g for 2 min. Subsequently, 800 µL of Mitochondria Isolation Reagent A was added. Vortex at medium speed for 5 s and incubate tube on ice for exactly 2 min. Transfer cell suspension to Dounce Tissue Grinder in ice and perform enough strokes to effectively lyse the cells. Next, return lysed cells to original tube and add 800 µL of Mitochondria Isolation Reagent C. Rinse Dounce Tissue Grinder with 200 µL of Reagent A and add to tube containing the sample. Centrifuge tube at 700 g for 10 min at 4°C. Transfer the supernatant to a new, 2.0 mL tube and centrifuge at 3,000 *g* for 15 min at 4°C. Transfer the supernatant (cytosol fraction) to a new tube. The pellet contains the isolated mitochondria. Boil mitochondrial pellet and cytosol fraction with SDS-PAGE sample buffer. Proteins were analysed by WB. Mitochondrial and cytoplasmic proteins expression was quantified and normalised to COX4I1 and ACTB (β-actin), respectively.

### 2.6 Western blotting assay

Proteins were resolved on Tris-glycine acrylamide gels followed by WB analysis. Primary antibodies included those against ACTB/β-Actin (ABclonal Technology, AC026; 1:200000), LC3-I/II (CST, 12741 1:1000), COX4I1 (Proteintech, 66110-1-Ig, 1:8000), PINK1 (CST, 6946, 1:1000), PRKN (CST, 4211, 1:1000), BAK1 (Proteintech, 29552-1-AP, 1:10000), BAX (Proteintech, 50599-2-lg, 1:10000), Ub (CST, 3,936, 1:1000), TNF-α (Proteintech, 60291-1-Ig, 1:8000), IL-1β (Proteintech, 16806-1-AP, 1:1000), IL-6 (Proteintech, 21865-1-AP, 1:1000). Detection was carried out using enhanced chemiluminescence assay and the densitometry analysis was performed using ImageJ software. Total proteins and cytoplasmic proteins expression was quantified and normalised to ACTB (β-actin), mitochondrial proteins expression was quantified and normalised to COX4I1.

### 2.7 Transmission electron microscopy (TEM)

Huh7 cells samples were prepared as previously described (Yao J. et al., 2022). The samples were observed under HT7700 TEM (Hitachi, Tokyo, Japan).

### 2.8 MitoTracker straining assay

Huh7 cells were stained with MitoTracker Red (Yeasen Biotech, 40741ES50) and MitoTracker Green (Yeasen Biotech, 40742ES50) according to the manufacturer’s instructions. Accordance with the instructions and acquired images using Cell Imaging Multi-Mode Reader (BioTek Cytarion1) driven by Gen5 software.

### 2.9 Immunoprecipitation (IP)

Cells lysates were incubated overnight with the corresponding antibodies at 4°C with gentle rotation. The primary antibodies included antibodies against PRKN (CST, 4211, 1:250), BAK1 (CST, 12105, 1:250), BAX (Proteintech, 50599-2-Ig, 1:250). Next day, immunogen-antibody complexes were captured by incubation with protein A/G magnetic beads (MedChem Express, HY-K0202) for 1 h at 4°C with gentle rotation. Then, the beads were washed 10 min with Pierce™ IP Lysis Buffer (Thermo, 87788) 3 times at 4°C. Immunocomplexes were analysed by WB.

### 2.10 Detection of MitoSOX generation

The MitoSOX measurement was conducted according to the manufacture protocol (Yeasen Biotech, 40778ES50). Accordance with the instructions and acquired images using Cell Imaging Multi-Mode Reader (BioTek Cytarion1) driven by Gen5 software.

### 2.11 Huh7 xenograft tumour models

Athymic nude female mice (4–6 weeks old) weighing 18–22 g was purchased from Beijing Vital River Laboratory Animal Technology Co., Ltd. (Certificate No. SCXK (JING)2021-0006). All procedures were approved by the Fourth Military Medical University Animal Care and Use Committee (NO.20220449). Huh7 cells (1×10^7^) were inoculated subcutaneously at the left forelimb pit of nude mice until tumour volume reached approximately 100 mm^3^. These tumour-bearing mice were grouped according to the tumour volume, with 6 mice in each group. Ailanthone was administered at a dose of 2 mg/kg via intraperitoneal injection (i.p.) once per day (The determination of animal experimental dose refers to previous literature reports (He et al., 2016)), 5-FU was administered at 15 mg/kg via i.p. once every 2 days. Mice were sacrificed under anesthesia after 21 days of treatment, the tumour xenografts were removed, photographed and weighed, and tumour tissues were used for IHC and IF assay. Tumour volume and mouse weight recorded twice a week, tumour volume was measured with vernier caliper, and calculated using the following formula: tumour volume (mm^3^) = D×d×d/2, in which D and d were the longest and the shortest diameters, respectively.

### 2.12 Immunohistochemical (IHC) staining

Samples preparation as previously described (Yao J. Y. et al., 2022). The primary antibodies included antibodies against TNF-α (Proteintech, 60291-1-Ig, 1:800), IL-1β (Proteintech, 16806-1-AP, 1:500), IL-6 (Proteintech, 21865-1-AP, 1:200), LC3 (Proteintech, 14600-1-AP, 1:500), PINK1 (Proteintech, 23274-1-AP, 1:4000), PRKN (Proteintech, 66674-1-Ig, 1:1000), BAK1 (Proteintech, 29552-1-AP, 1:500), BAX (Proteintech, 50599-2-lg, 1:4000).

### 2.13 Immunofluorescence (IF) and confocal microscope analysis

Huh7 cells and tumour tissue samples preparation as previously described (Yao J. et al., 2022). The fluorescent spot pixel value was used to quantify the fluorescent images. The primary antibodies as follows: PINK1 (Proteintech, 23274-1-AP, 1:500), PRKN (Proteintech, 66674-1-Ig, 1:100; Proteintech, 14060-1-AP, 1:200), BAK1 (Proteintech, 29552-1-AP, 1:200), BAX (Proteintech, 60267-1-Ig, 1:200).

### 2.14 Enzyme-linked immunosorbent assay (ELISA)

Cell supernatant levels of these proinflammatory cytokines measured by ELISA using commercially available kits (TNF-α, Thermo, 88-7346-86; IL-1β, Thermo, 88-7261-86; IL-6, Thermo, 88-7066-86) and expressed as picograms per millilitre.

### 2.15 QPCR analysis

Total RNA was extracted using Total RNA Extractor (Sangon Biotech, B511311) and then amplified by PCR. A 1 μg aliquot of total RNA was used to transcribe first-strand cDNA with a HiScript II Q RT SuperMix for qPCR (Vazyme BioTech, R223). For detected cytosolic and mitochondrial mtDNA, mitochondrial and cytoplasm fractions extraction was performed with a Mitochondria Isolation Kit for Cultured Cells (Thermo, 89874) according to manufacturer’s instructions. Cytosolic and mitochondrial total DNA was isolated using FastPure Cell/Tissue DNA Isolation Mini Kit (Vazyme BioTech, DC102-01). QPCR was completed on an ABI PRISM Sequence Detector 7500 (PerkinElmer, Branchburg, NJ) using Sequence Detector version 1.7 software (Applied Biosystems, Foster City, CA). SYBR Green PCR Master Mix was purchased from Vazyme BioTech (Q131-02/03). The primers used for PCR amplification were as follows:

*LC3*-sense: 5′-GAT​GTC​CGA​CTT​ATT​CGA​GAG​C-3′
*LC3*-antisense: 5′-TTG​AGC​TGT​AAG​CGC​CTT​CTA-3′
*PINK1*-sense: 5′-GGA​GGA​GTA​TCT​GAT​AGG​GCA​G-3′
*PINK1*-antisense: 5′-AAC​CCG​GTG​CTC​TTT​GTC​AC-3′
*PRKN*-sense: 5′-GTG​TTT​GTC​AGG​TTC​AAC​TCC​A-3′
*PRKN*-antisense: 5′-GAA​AAT​CAC​ACG​CAA​CTG​GTC-3′
mtDNA gene-*Cytochrome c oxidase I* -sense: 5′-GCC​CCA​GAT​ATA​GCA​TTC​CC-3′
mtDNA gene-*Cytochrome c oxidase I* -antisense: 5′-GTT​CAT​CCT​GTT​CCT​GCT​CC-3′
mtDNA internal reference gene*-18S rDNA*-sense: 5′-TAG​AGG​GAC​AAG​TGG​CGT​TC-3′
mtDNA internal reference gene*-18S rDNA*-antisense: 5′-CGC​TGA​GCC​AGT​CAG​TGT-3′

### 2.16 Statistical evaluation

The data are presented as mean ± SD from triplicate parallel experiments unless otherwise indicated. Statistical analyses were performed using one-way ANOVA, with *P* values < 0.05 being considered significant. Significance levels *p < 0.05 and **p < 0.01 were considered as relatively significant levels.

## 3 Results

### 3.1 Ailanthone inhibits proliferation and induces mitochondrial dysfunction of HCC cells

**FIGURE 1:**
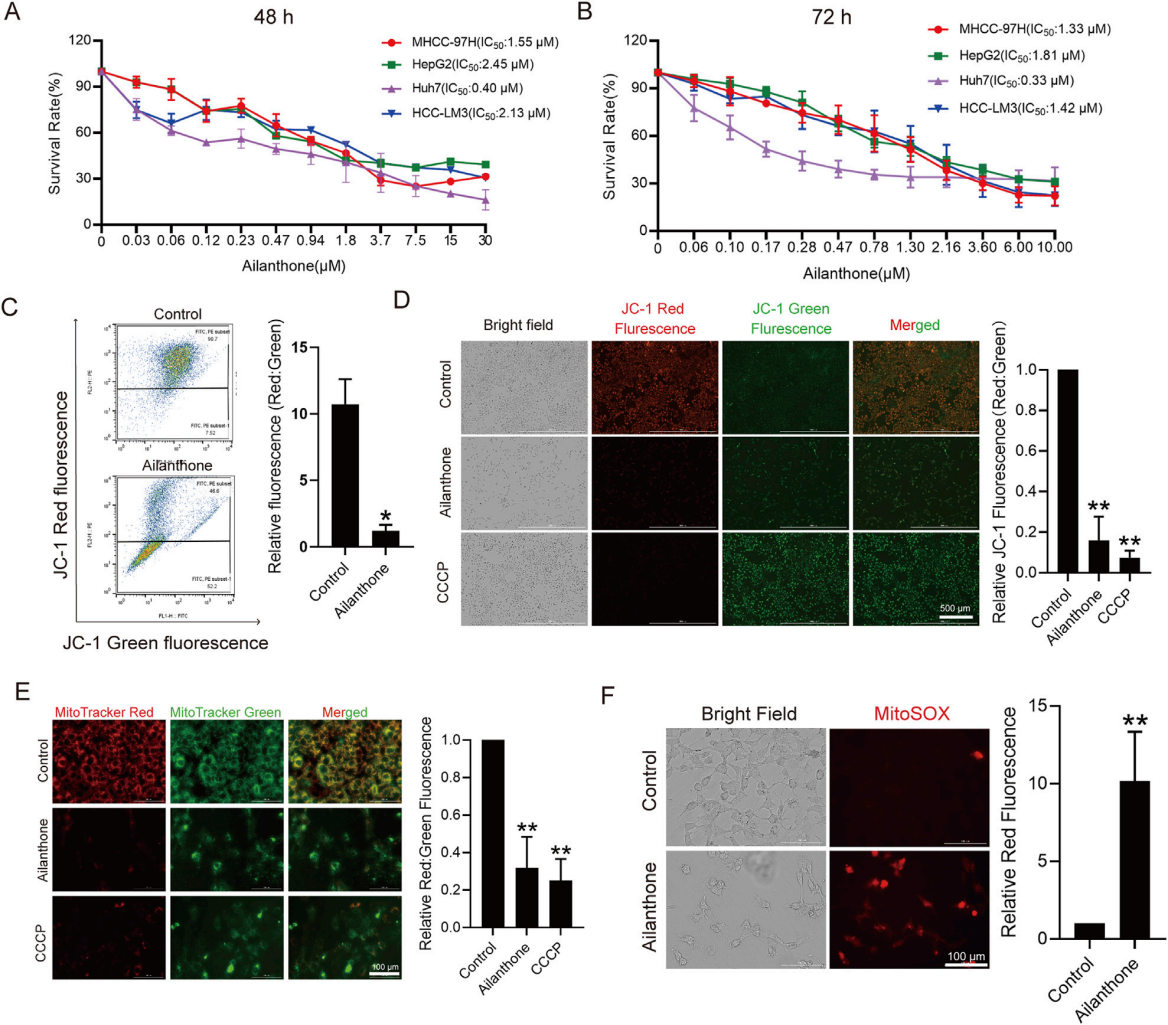
Ailanthone inhibits proliferation and induces mitochondrial dysfunction of HCC cells. **(A, B)** CCK8 assay measured HCC cells viability after ailanthone treated for 48 h and 72 h. **(C, D)** Huh7 were treated with 1.2 μM ailanthone for 48 h, or 10 μM CCCP for 24 h, MMP were measured using MitoProbe JC-1 stain by flow cytometry **(C)** and fluorescence imaging (40×, scale bar: 500 μm) **(D)**. **(E)** MitoTracker Red and MitoTracker Green staining was measured by fluorescence imaging (200×, scale bar: 100 μm). The ratio of red to green fluorescence intensity was quantified. **(F)** Levels of MitoSOX was measured by MitoSOX stain and observed by fluorescence intensity (200×, scale bar: 100 μm). *Bar, SD*. *p < 0.05 or **p < 0.01.

We investigated the effects of ailanthone on the proliferation of four HCC cell lines, MHCC-97H, HepG2, Huh7 and HCC-LM3, using the CCK8 assay. Ailanthone drastically reduced the proliferation of both HCC cell lines, especially Huh7 cells, with IC_50_ values of 0.40 μM and 0.33 μM at exposure durations of 48 h and 72 h, respectively (Figures 1A, B). Therefore, Huh7 cells were used for further evaluation.

Given the critical role of mitochondrial homeostasis in HCC cell proliferation, we assessed mitochondrial membrane potential (MMP) using JC-1 staining. As shown in Figure 1C, flow cytometry results indicated that the ratio of JC-1 J-aggregates to monomers (Red: Green fluorescence) decreased by 88.2% (*p* = 1.99e-02) after treated with ailanthone. Consistent with the flow cytometry results, fluorescence imaging showed that ailanthone reduced the ratio of JC-1 J-aggregates to monomers (Red: Green fluorescence) by 84.0% (*p* = 2.37e-04) (Figure 1D). These results indicated that ailanthone induced MMP loss (Figures 1C, D), with CCCP serving as a positive control (Figure 1D). In addition, the mitochondrial fluorescent dye MitoTracker Red is dependent on MMP, whereas MitoTracker Green is MMP-independent. Therefore, we further calculated the fluorescence ratio of MitoTracker Red and Green to determine total mitochondrial activity under ailanthone treated. Results showed that ailanthone significantly reduced the fluorescence ratio of MitoTracker Red to Green by 68.1% (*p* = 2.02e-03) (Figure 1E), suggesting MMP loss, consistent with the JC-1 staining results. Mitochondrial reactive oxygen species (MitoSOX) is another important indicator of mitochondrial activity, and increased oxidative stress triggers mitochondrial damage (Wang et al., 2022). We observed that ailanthone also increased MitoSOX generation (Figure 1F). Collectively, these results indicated that ailanthone inhibited the proliferation of hepatoma cell lines and induced mitochondrial dysfunction.

### 3.2 Ailanthone blocks mitochondrial turnover by suppressing mitophagy

**FIGURE 2:**
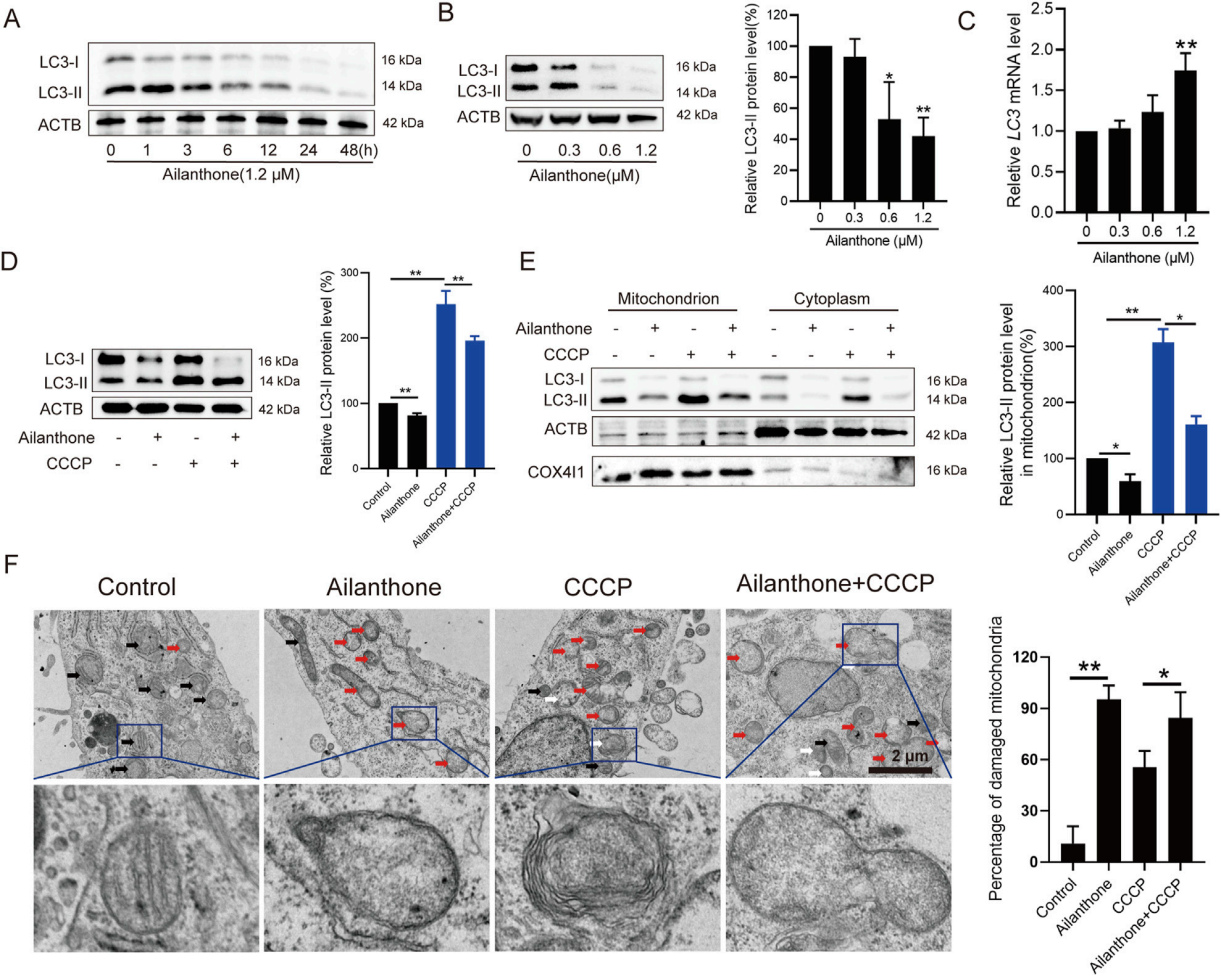
Ailanthone blocks mitochondrial renewal by suppressing mitophagy. **(A, B)** WB assays were performed to assess the LC3-I/II protein expression in Huh7 cells treated with ailanthone (1.2 μM) for different times **(A)**, and treated with ailanthone for 48 h **(B)**. Proteins expression was quantified and normalised to ACTB (β-actin). **(C)** Ailanthone treated Huh7 cells for 48 h, *LC3* mRNA levels were detected by qPCR assay. **(D, E)** Huh7 cells were treated with 1.2 μM ailanthone for 48 h with or without 10 μM CCCP for 24 h. The protein levels of LC3-I/II in whole cells **(D)**, mitochondria and the cytoplasm were measured, the protein level of the LC3-II form in mitochondria was quantified and normalised to COX4I1 **(E)**. **(F)** Detection of mitochondrial ultrastructure by TEM. Black arrow: normal mitochondria. White arrow: mitochondrial autophagosome. Red arrow: damaged mitochondria (mitochondria with swelling, broken cristae and vacuolation). The percent damaged mitochondria were quantified (5000×, scale bar: 2 μm). *Bar, SD.* *p < 0.05 or **p < 0.01.

Normally, dysfunctional mitochondria initiate mitophagy to promote mitochondrial turnover and maintain mitochondrial homeostasis, enabling cancer cells to better adapt to aggressive conditions (Lu et al., 2023). However, we observed that ailanthone reduced autophagosome marker LC3-II expression in a time- and concentration-dependent manner (Figures 2A, B). Nevertheless, it had no significant effect on suppressing the transcription of the *LC3* gene (Figure 2C). It is possible that ailanthone downregulated LC3 protein levels through a post-transcriptional mechanism. Under treatment with 1.2 µM ailanthone, due to the strong decrease in LC3 protein levels, a negative feedback mechanism might be activated to enhance the *LC3* gene transcription. However, due to the presence of ailanthone, this upregulation might still fail to effectively translate into an increase in protein levels. CCCP is a mitophagy inducer (Georgakopoulos et al., 2017). We found that ailanthone reduced the CCCP-induced accumulation of LC3-II in HCC cells (Figure 2D). Thus, we hypothesized that ailanthone induced mitochondrial dysfunction in HCC cells while suppressing mitophagy. To further validate the inhibitory effect of ailanthone on mitophagy, we analyzed LC3-II protein expression in mitochondria. WB assay showed that ailanthone inhibited the accumulation of LC3-II on mitochondria, while mitophagy inducer CCCP significantly promoted the accumulation of LC3-II protein in mitochondria, ailanthone still attenuated the effect of CCCP (Figure 2E). These results suggesting that ailanthone inhibited mitophagy. Subsequently, we detected the ultrastructure of the mitochondria using TEM. We observed that ailanthone resulted in a large accumulation of damaged mitochondria in cells, with mitochondrial ridges largely dissolved, and the mitochondria were generally swollen and vacuolated (marked with red arrows). The CCCP-treated group displayed largely dissolved mitochondrial ridges and increased mitophagy (marked with white arrows). Moreover, cotreatment with ailanthone and CCCP reduced CCCP-induced mitophagy (Figure 2F). These results suggest that ailanthone inhibited mitophagy, resulting in the disruption of mitochondrial homeostasis.

### 3.3 Ailanthone suppresses mitophagy by disturbing the PINK1-PRKN axis

**FIGURE 3:**
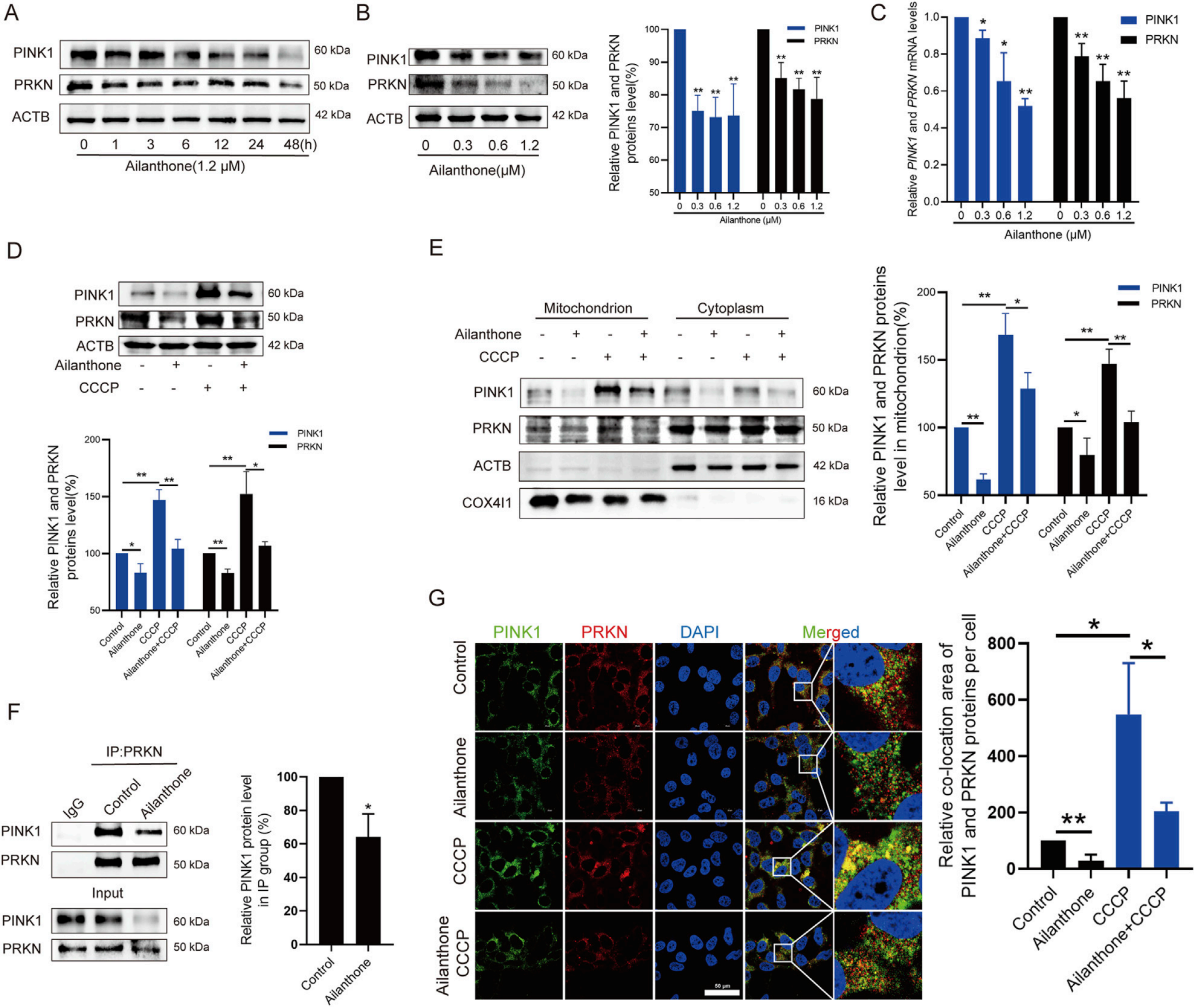
Ailanthone suppresses mitophagy by disturbing PINK1-PRKN axis. **(A, B)** WB assays were performed to assess the PINK1 and PRKN proteins expression in Huh7 cells treated with ailanthone (1.2 μM) for different times **(A)**, and treated with ailanthone for 48 h **(B)**. **(C)** Huh7 cells were treated by ailanthone for 48 h, *PINK1* and *PRKN* mRNA levels were detected by qPCR assay. **(D, E)** Huh7 cells were treated with 1.2 μM ailanthone for 48 h with or without CCCP (10 μM for 24 h), the proteins expression of PINK1 and PRKN in whole cells **(D)**, and in mitochondria and cytoplasm **(E)** were measured by WB assay. **(F)** Immunoprecipitated against PRKN, and WB assays for PINK1 and PRKN were performed. Quantitative analysis of PINK1 expression normalised to that of PRKN in the IP group. **(G)** Huh7 cells were treated with 1.2 μM ailanthone for 48 h with or without CCCP (10 μM for 24 h). The colocalization of PINK1 with PRKN was observed by confocal microscopy (1000×, scale bar: 50 μm). The colocalization area of PINK1 with PRKN per cell was quantified. *Bar, SD*. *p < 0.05 or **p < 0.01.

To date, mitophagy induction is mainly attributed to the PINK1-PRKN axis (Pickrell and Youle, 2015). Depolarized mitochondria recruit PINK1 and the E3 ubiquitin ligase PRKN, which ubiquitinates mitochondrial membrane proteins and mediates mitophagy (Soutar et al., 2019). WB assay revealed that ailanthone suppressed PINK1 and PRKN expression in a time- and concentration-dependent manner (Figures 3A, B). At the same time, *PINK1* and *PRKN* mRNA levels were decreased by ailanthone (Figure 3C). Therefore, we hypothesised that ailanthone suppressed mitophagy by blocking the PINK1-PRKN pathway. CCCP, which serves as a mitophagy-stimulating chemical tool, can lead to PINK1-PRKN-dependent mitophagy (Georgakopoulos et al., 2017). As shown in Figure 3D, CCCP strongly activated PINK1 and PRKN proteins, whereas ailanthone attenuated the effect of CCCP in HCC cells. To further validate the inhibitory effect of ailanthone on the PINK1-PRKN axis, we investigated the PINK1 and PRKN proteins level on mitochondria. The results showed that ailanthone significantly inhibited PINK1 and PRKN proteins accumulation on mitochondria, and weakened CCCP-induced accumulation of PINK1 and PRKN (Figure 3E). Moreover, ailanthone disturbed the proteins binding between PINK1 and PRKN (Figure 3F). The immunofluorescence (IF) assay showed ailanthone reduced PINK1 and PRKN proteins colocalization (Figure 3G). In general, these results demonstrated that ailanthone suppressed mitophagy by disturbing the PINK1-PRKN axis.

### 3.4 Ailanthone aggravates mtDNA-induced inflammation

**FIGURE 4:**
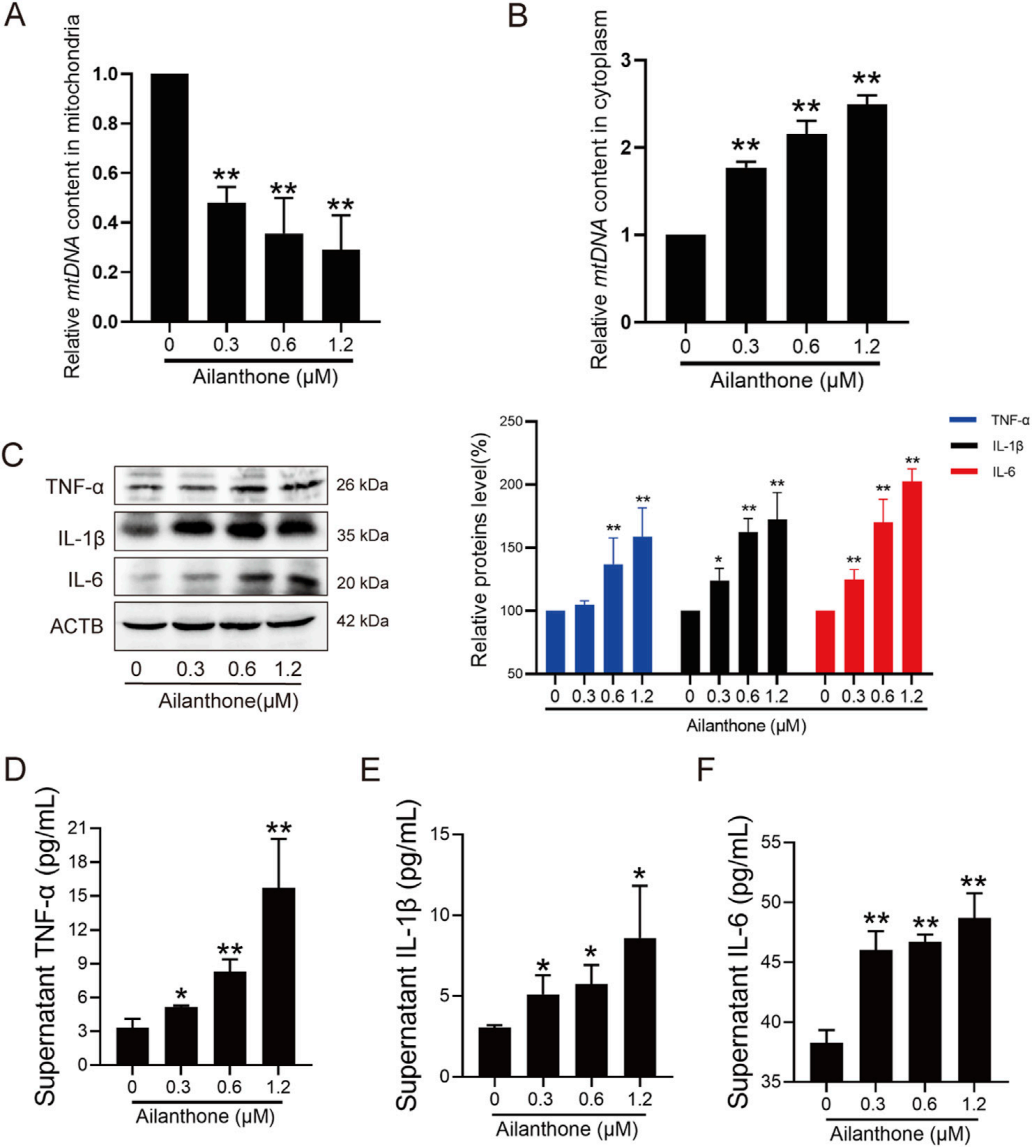
Ailanthone aggravates mtDNA-induced inflammation. **(A, B)** QPCR was performed to detect mtDNA contents in mitochondrial and cytoplasmic components after Huh7 cells were treated with ailanthone for 48 h. **(C–F)** Huh7 cells were treated with ailanthone for 48 h, WB assays were performed to assess the TNF-α, IL-1β and IL-6 proteins expression, proteins expression was quantified and normalised to ACTB **(C)**, and ELISA assay measured cytokines TNF-α, IL-1β and IL-6 production in medium supernatant **(D–F)**. *Bar, SD*. *p < 0.05 or **p < 0.01.

Mitophagy plays an important role in controlling inflammatory responses (Marchi et al., 2023). In some cases, the leakage of mtDNA from damaged mitochondria elicits inflammation and stimulates immunostimulatory cytokine production (Ghiringhelli et al., 2009; Marchi et al., 2023). The lack of PINK1 and PRKN leads to mtDNA leakage into the cytoplasm (Sliter et al., 2018). Therefore, we further investigated whether the anti-HCC effect of ailanthone was related to mtDNA leakage elicited inflammation response. We first assessed the mtDNA content separately in the mitochondrial and cytoplasmic fractions. The qPCR results indicated that mtDNA contents decreased in the mitochondrial fraction and increased in the cytosolic fraction following treatment with ailanthone (Figures 4A, B), indicating that high levels of ailanthone-induced mtDNA were released into the cytoplasm.

The inflammatory cytokine tumour necrosis factor α (TNF-α) is central in orchestrating the inflammatory immune response (van Loo and Bertrand, 2023). We observed that the TNF-α protein level was significantly upregulated by ailanthone (Figure 4C). Further examination revealed that inflammatory cytokines interleukin-1β (IL-1β) and interleukin-6 (IL-6) were also upregulated by ailanthone (Figure 4C). ELISA revealed that high levels of TNF-α, IL-1β, and IL-6 were released into the cell supernatant (Figures 4D–F), suggesting activation of the inflammatory response. Therefore, these results indicated that ailanthone induced mtDNA leakage from dysfunctional mitochondria, triggering an inflammatory response.

### 3.5 Ailanthone reduces PRKN-mediated BAX degradation and promotes BAX-BAK1 mitochondrial pores formation

**FIGURE 5:**
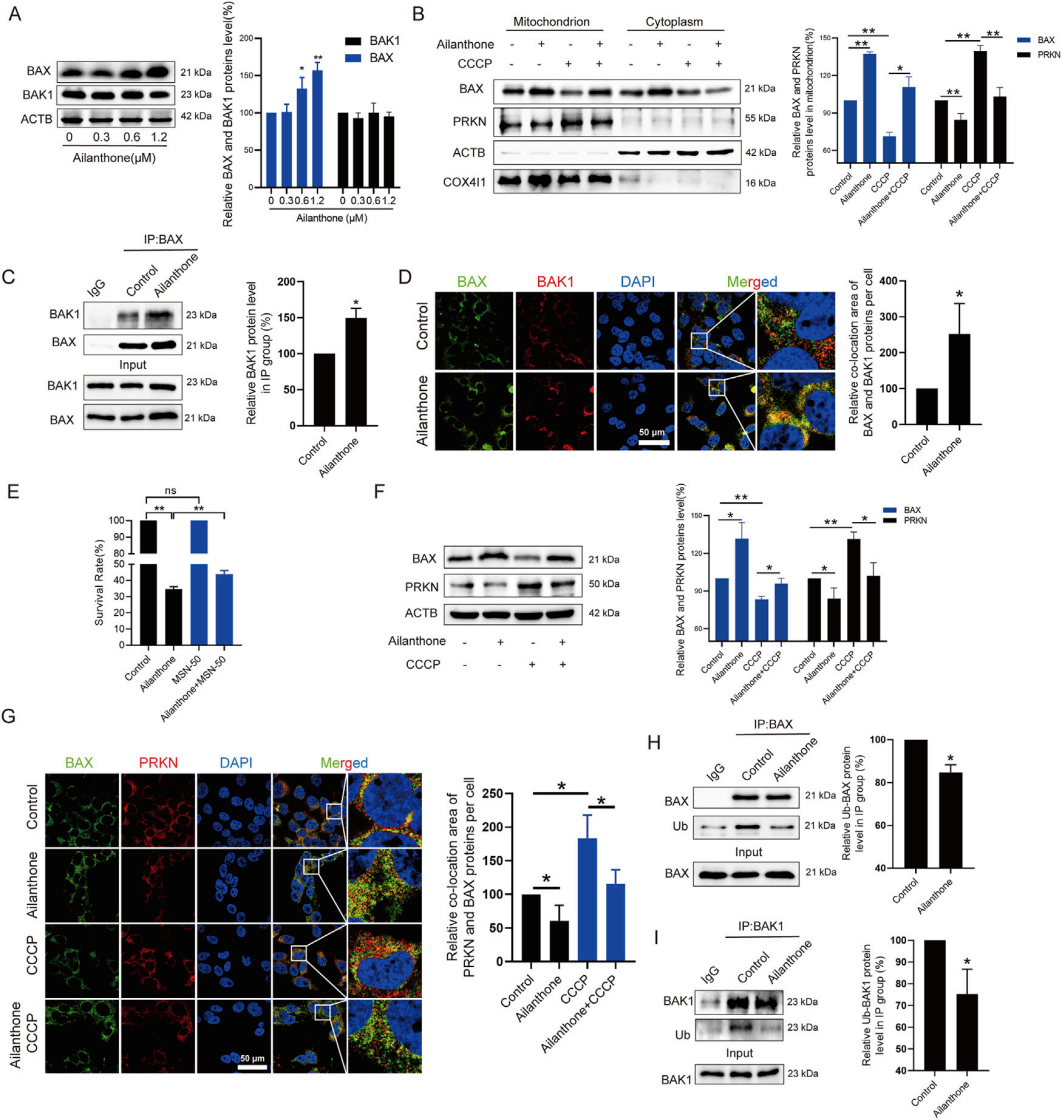
Ailanthone reduces PRKN-mediated BAX degradation and promotes BAX-BAK1 mitochondrial pores formation. **(A)** WB assays were performed to assess the BAX and BAK1 proteins expression in Huh7 cells treated with ailanthone for 48 h. **(B)** Huh7 cells were treated with 1.2 μM ailanthone for 48 h with or without CCCP (10 μM for 24 h), BAX and PRKN proteins expression in mitochondria and cytoplasm were measured by WB. **(C, D)** Huh7 cells were treated with 1.2 μM ailanthone for 48 h. The lysates were immunoprecipitated against BAX, and immunoblotting assays for BAX and BAK1 were performed **(C)**. The colocalization of BAX and BAK1 was observed (1000×, scale bar: 50 μm). The colocalization area of BAX with BAK1 per cell was quantified **(D)**. **(E)** Huh7 cells were treated with MSN-50 (1 μM for 3 h), MSN-50 was then removed, and the cells were treated with 0.6 μM ailanthone for 48 h. CCK8 assay measured cell viability. **(F, G)** Huh7 cells were treated with 1.2 μM ailanthone for 48 h with or without CCCP (10 μM for 24 h), BAX and PRKN proteins expression were measured by WB **(F)**. The colocalization of BAX and PRKN proteins were observed (1000×, scale bar: 50 μm). The colocalization area of BAX with PRKN per cell was quantified **(G)**. **(H, I)** IP analysis of BAX and BAK1 in Huh7 cells treated with 1.2 μM ailanthone for 48 h was conducted. The ubiquitinated modified BAX and BAK1 signals were visualized using a pan-ubiquitin antibody. The quantitative analysis of ubiquitinated BAX and BAK1 proteins expression was normalized to their respective levels in the IP group. *Bar, SD.* *p < 0.05 or **p < 0.01.

MtDNA leakage is associated with BAX-BAK1 mitochondrial pores formation (Marchi et al., 2023). Under normal conditions, BAX localises to the cytosol; however, during apoptosis, it translocates to the mitochondria to form BAX-BAK1 mitochondrial pores that facilitate mtDNA leakage, subsequently triggering an inflammatory response (McArthur et al., 2018). Therefore, we subsequently explored whether ailanthone affected the BAX-BAK1 mitochondrial pores formation. WB assay showed that ailanthone upregulated BAX protein without affecting the BAK1 protein level (Figure 5A). Furthermore, ailanthone significantly promoted BAX protein translocated to the mitochondria (Figure 5B). Immunoprecipitation (IP) revealed that ailanthone increased the binding of BAX and BAK1 proteins (Figure 5C). IF results showed that ailanthone increased the BAX and BAK1 proteins colocalization (Figure 5D). Conversely, pretreatment with BAX-BAK1 oligomerization inhibitor MSN-50 (Niu et al., 2017) weakened the ailanthone’s anti-proliferation effect in Huh7 cells (Figure 5E). Hence, we concluded that ailanthone promoted BAX-BAK1 mitochondrial pores formation.

PRKN directly ubiquitinated the BAX and BAK1 proteins and promoted BAX degradation, but does not promote BAK1 degradation (Cakir et al., 2017; Bernardini et al., 2019). Ubiquitinated BAK1 was not targeted for proteasomal degradation (Bernardini et al., 2019). We have demonstrated that ailanthone inhibited the PINK1-PRKN axis and increased BAX protein expression. Therefore, we next investigated whether ailanthone increased BAX protein level by inhibiting PRKN. As Figure 5F showed that ailanthone inhibited PRKN protein while upregulating BAX protein level, when CCCP activated PRKN protein, the expression of BAX protein decreased, and ailanthone weakened the inhibitory effect of CCCP on BAX. Furthermore, ailanthone inhibited PRKN localization on mitochondria while increasing BAX’s accumulation there. Conversely, CCCP induced PRKN localization on mitochondria but reduced BAX localization on mitochondria. Ailanthone reduced the regulatory effect of CCCP on BAX (Figure 5B). IF assay showed that ailanthone reduced PRKN and BAX proteins colocalization, and weakened the CCCP-induced colocalization of PRKN and BAX (Figure 5G). Furthermore, IP assay revealed that ailanthone promoted BAX and BAK1 proteins deubiquitination (Figures 5H, I). Therefore, we concluded that ailanthone promoted the deubiquitination of BAX by inhibiting PRKN, ultimately increasing BAX protein level. Although deubiquitylation of BAK1 by ailanthone did not affect its protein level, it might be also another critical pathway for regulating HCC proliferation (Bernardini et al., 2019). Collectively, these results indicated that ailanthone reduced PRKN-mediated BAX ubiquitination degradation and enhanced BAX-BAK1 mitochondrial pores formation.

### 3.6 Ailanthone inhibits Huh7 xenograft model growth by blocking PINK1-PRKN-mediated mitophagy and promoting BAX-BAK1 mitochondrial pores formation

**FIGURE 6:**
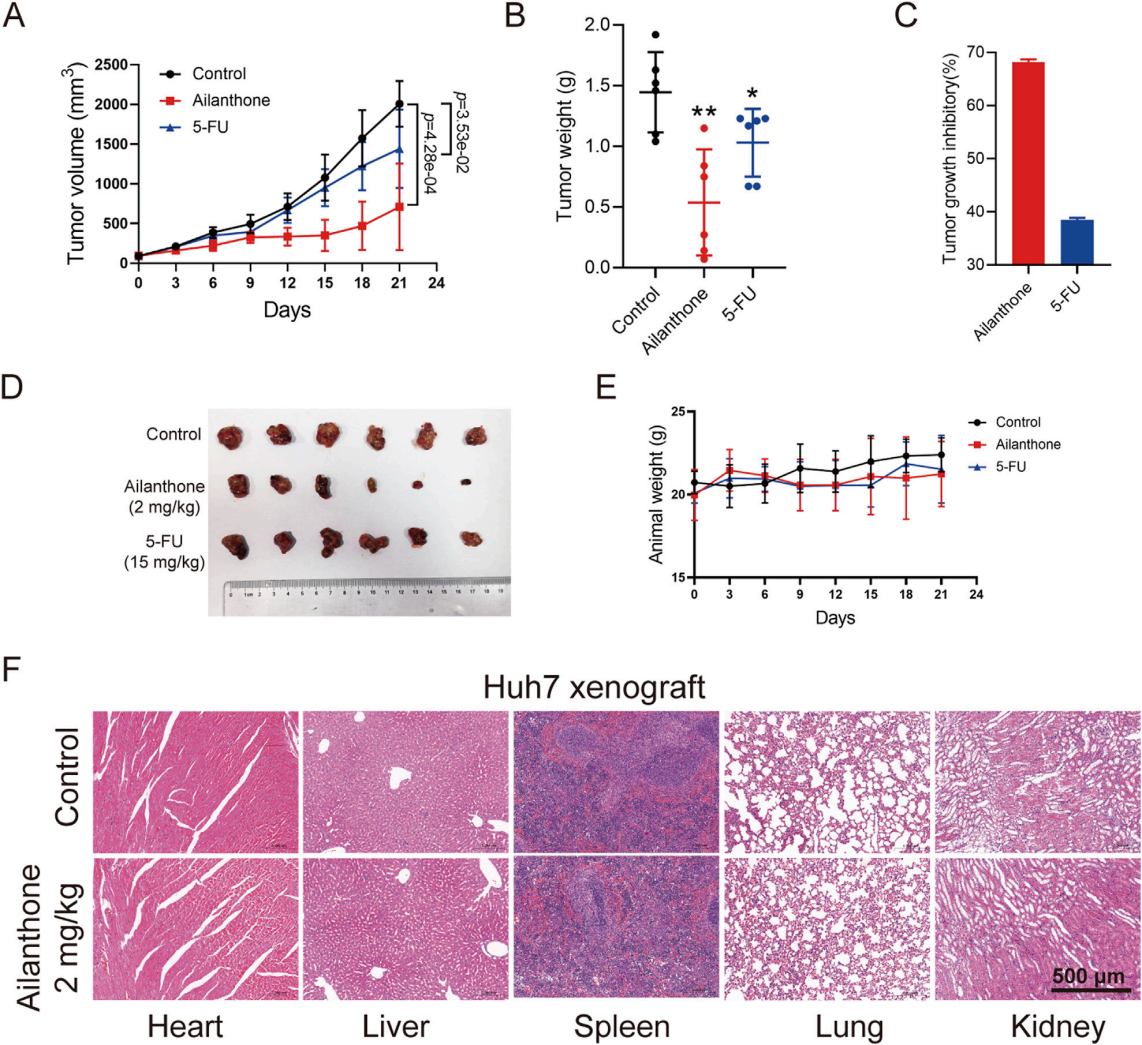
Ailanthone inhibits the hepatoma growth of Huh7 xenograft model. Huh7 cells xenograft mouse models were treated with physiological saline, ailanthone (2 mg/kg, i.p., once per day) and 5-FU (15 mg/kg, i.p., once every 2 days). Tumour volume and mouse weight recorded twice a week. Mice were sacrificed under anesthesia after 21 days of treatment, the tumour xenografts were removed, photographed and weighed. **(A)** The tumour volumes. **(B)** Tumour weight. **(C)** Inhibition rates of tumour weight. **(D)** Image of tumour. **(E)** Changes in mouse weight. **(F)** Histological analysis of heart, liver, spleen, lung and kidney (100×, scale bar: 500 μm). *Bar, SD.* *p < 0.05 or **p < 0.01.

Previous study proved that oral administration of ailanthone (5 mg/kg) resulted in moderate weight loss and significant gastric injury in mice, while intraperitoneal injection (i.p.) of ailanthone (2 mg/kg) did not show toxicity (He et al., 2016), and 2 mg/kg dose of ailanthone demonstrated the best results in inhibiting tumour growth and improving survival rates in non-small cell lung cancer and prostate cancer (He et al., 2016; Fang et al., 2024). Based on these findings, we chose i.p. of 2 mg/kg as the *in vivo* study dose of ailanthone for anti-HCC. We established Huh7 xenograft model. Ailanthone was administered at a dose of 2 mg/kg via i.p. once per day, 5-Fluorouracil (5-FU) was administered at 15 mg/kg via i.p. once every 2 days. Tumour volume and mouse weight recorded twice a week. Mice were sacrificed under anesthesia after 21 days of treatment, the tumour xenografts were removed, photographed and weighed. We found that the tumour volume in the ailanthone-treated group was significantly lower than that in the vehicle group (Figure 6A). Furthermore, ailanthone reduced the tumour weight with a tumour inhibitory rate of 68.14%, which was higher than 5-FU (Figures 6B–D). Notably, ailanthone exhibited potent antitumour activity *in vivo* without affecting the body (Figure 6E). Moreover, HE staining revealed no apparent changes in the physiological functions of vital organs in the ailanthone-treated group (Figure 6F). Therefore, ailanthone inhibited HCC growth *in vivo* with low toxicity.

**FIGURE 7:**
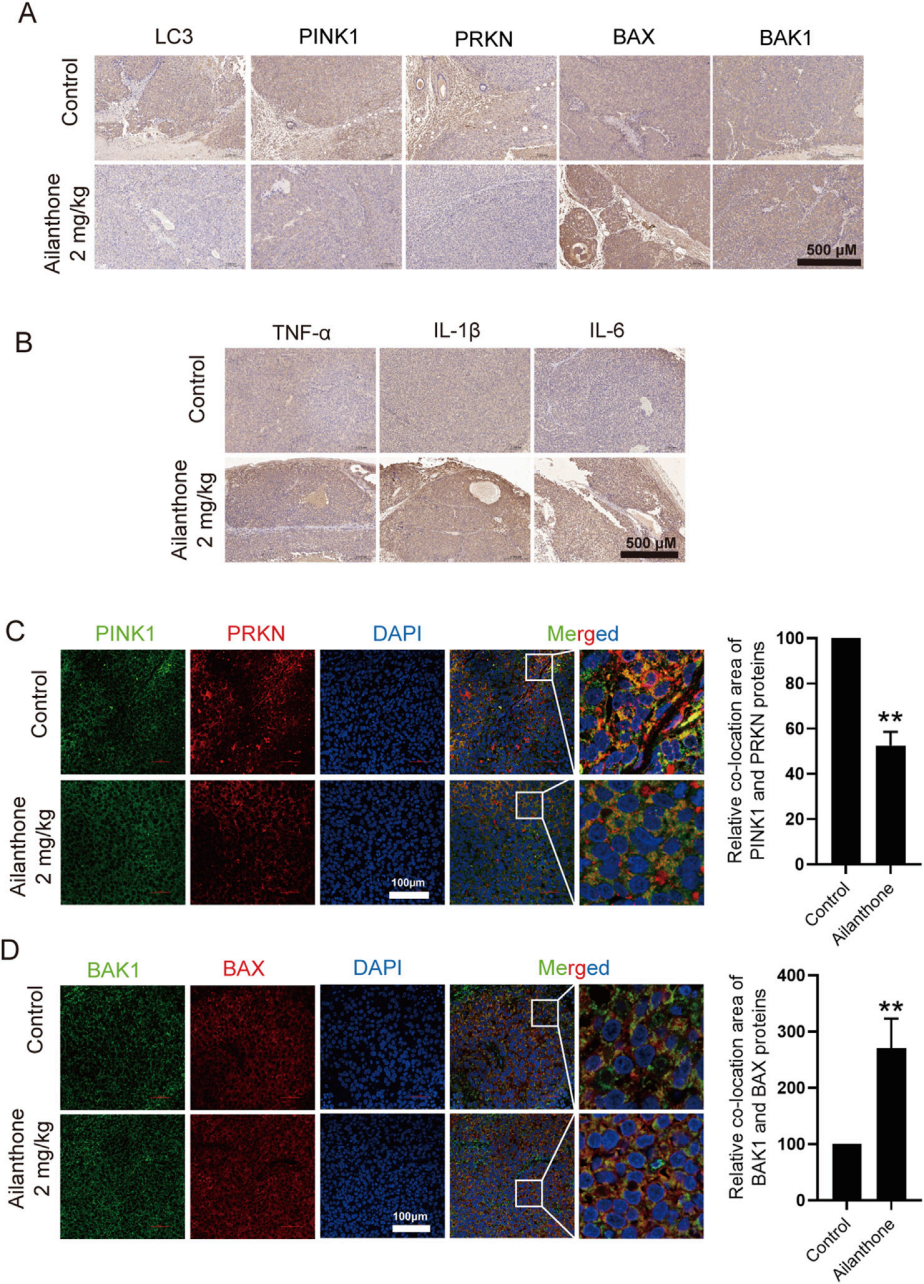
Ailanthone blocks PINK1-PRKN-mediated mitophagy while promoting BAX-BAK1 mitochondrial pores formation and inducing inflammation *in vivo*. **(A, B)** IHC detection of LC3, PINK1, PRKN, BAX, BAK1, TNF-α, IL-1β, and IL-6 proteins expression in tumour tissues (100×, scale bar: 500 μm). **(C, D)** The colocalization of PINK1 with PRKN and BAX with BAK1 in tumour tissues was observed by confocal microscopy imaging (400×, scale bar: 100 μm). The colocalization areas were quantified. *Bar, SD.* **p < 0.01.

To further elucidate the molecular mechanism underlying the effect of ailanthone against HCC *in vivo*, we performed IHC analysis to investigate the protein expression of LC3, PINK1, PRKN, BAX, and BAK1 in tumour tissues. Ailanthone inhibited the expression of LC3, PINK1, and PRKN while upregulating that of BAX, with less effect on BAK1 expression (Figure 7A). Furthermore, ailanthone induced the expression of the inflammatory factors TNF-α, IL-1β, and IL-6 in tumour tissues (Figure 7B). Tissue fluorescence assays demonstrated that ailanthone reduced the colocalization of PINK1 with PRKN and increased BAX with BAK1 (Figures 7C, D), which were consistent with our observations *in vitro*. Hence, ailanthone inhibited PINK1-PRKN-mediated mitophagy, reduced BAX degradation, and promoted BAX-BAK1 mitochondrial pores formation, thereby aggravating inflammation *in vivo*. Collectively, these results suggest that ailanthone is an effective agent with a potent antitumour activity and a favourable safety profile *in vivo*.

## 4 Discussion

Inhibiting mitophagy is a suggested therapeutic strategy for promoting cancer cell death. For example, oroxylin A from *Scutellaria baicalensis* exhibits strong therapeutic potential against HCC by downregulating PINK1-PRKN-mediated mitophagy (Yao J. et al., 2022). Tanshinone IIA inhibits mitophagy and enhances apoptosis in colorectal cancer cells by inhibiting the PRKN pathway (He and Gu, 2018). Targeting mitophagy is also frequently used to enhance drug sensitivity in cancer therapy. PINK1-mediated mitophagy confers cancer cells with the ability to survive chemotherapy, whereas inhibiting mitophagy restores chemosensitivity in chemotherapy-treated ESCC cells (Yamashita et al., 2017). In addition, a combination of cisplatin and the mitophagy inhibitor PKI-402 significantly enhances the sensitivity of HCC cells to cisplatin (Sheng et al., 2019). In this study, we found that ailanthone exhibited potent antiproliferative ability against HCC by blocking PINK1-PRKN-mediated mitophagy, warranting further research into the combination of ailanthone and chemotherapeutic drugs. Nevertheless, combination therapy also has risks and limitations, including drug interactions, toxicity concerns, and the risk of accelerated drug resistance. To thoroughly explore the possibility of its combination therapy, further research is need to validate its efficacy in more HCC models, such as primary cancer models and patient-derived xenograft (PDX) models. Pharmacokinetic/pharmacodynamic analysis and toxicity evaluation of combination therapy efficacy are also necessary.

CCCP is a potent mitochondrial oxidative phosphorylation uncoupling agent, which exacerbates PINK1-dependent mitophagy by uncoupling the mitochondrial proton gradient (Georgakopoulos et al., 2017). It is commonly used as a positive control for MMP loss and PINK1-PRKN mediated mitophagy (Di Rienzo et al., 2022; Yao J. Y. et al., 2022; Yi et al., 2024). Our study showed that ailanthone inhibited CCCP induced LC3 protein aggregation and PINK1-PRKN-mediated mitophagy. However, the PINK1-PRKN mediated mitophagy induced by CCCP is due to mitochondrial respiratory chain damaged, and CCCP can also induce cell apoptosis (de Graaf et al., 2004; Kane et al., 2018). Therefore, when ailanthone and CCCP are combined, ailanthone inhibited CCCP-induced mitophagy, however, the resultant antitumor effects are additive, antagonistic, or independent require further investigation.

Mitophagy plays a crucial role in minimising reactive oxygen species generation, thereby inhibiting the activation of intracellular proinflammatory factors, including the NLRP3 inflammasome and NF-κB, by preserving the functional pool of mitochondria (Brenner et al., 2013). During mitochondrial dysfunction, mtDNA is released into the cytosol, activating the NLRP3 inflammasome and triggering CASP1 activation, which in turn leads to the proteolytic maturation of IL-1β and IL-18 (Marchi et al., 2023). It is reported that the cytosolic mtDNA also induced inflammation by activating the cGAS-STING pathway. The activated cGAS synthesizes cGAMP from GTP and ATP, which serves as a second messenger. cGAMP then activates STING located on the surface of the endoplasmic reticulum (ER). STING, in turn, activates the transcription factors IRF3 and NF-κB, which induces the inflammatory cytokines expression (Li and Chen, 2018). Of note, inflammation plays a dual role in cancer, enhancing immune responses to combat tumors while also potentially promoting tumorigenesis in some contexts, largely depending on the intensity and duration of inflammation (Marchi et al., 2023). For instance, chronic inflammation was associated with oncogenesis and accelerated tumour progression. Nevertheless, in tumour therapy, potent inflammatory responses culminating in the engagement of adaptive immunity underlie the beneficial effects of numerous cancer therapies, including conventional chemotherapeutics, targeted anticancer agents, and radiotherapy (Marchi et al., 2023). In the present study, we have demonstrated that ailanthone significantly activates inflammatory cytokines, triggered by mtDNA leakage from damaged mitochondria. Cytosolic mtDNA also widely recognized as cellular inner immune sensors (Cai et al., 2014). It plays a crucial role in facilitating the secretion of cGAS-STING1-dependent type I interferons, which have an important role in the differentiation of both CD8^+^T cells, CD4^+^T cells and NK^+^ cells, as well as in immune-mediated suppression of tumour growth (Yu P. et al., 2022; Saha et al., 2024). The binding of programmed cell death protein ligand 1 (PD-L1) to programmed cell death protein 1 (PD-1) leads to the blockade of immune cell activity and impairs T-cell-mediated immune responses, often resulting in the failure of tumour immunotherapy. (Wang et al., 2019). Notably, ailanthone suppresses PD-L1 transcription through the ailanthone-c-Jun-PD-L1 pathway in melanoma cells, thereby blocking PD-L1 secretion (Yu P. et al., 2022). Consequently, there is potential for ailanthone to inhibit HCC by eliciting tumour-targeting immune responses, which merits further investigation.

PRKN is a key regulator of mitophagy that promotes the recovery of intracellular homeostasis by increasing the ubiquitination-dependent inactivation of BAK1 and BAX (Bock and Tait, 2020). The proapoptotic pore-forming proteins BAX and BAK1 in the outer membrane of mitochondria enable the extrusion of the inner mitochondrial membrane into the cytosol, culminating in its breakdown and subsequent mtDNA leakage, ultimately causing inflammatory reactions (McArthur et al., 2018). BAX and BAK1 also initiate mitochondrial outer membrane permeabilization (MOMP), which is a key step in at least two types of caspase-dependent regulated cell death, including intrinsic apoptosis and extrinsic apoptosis in type II cells (Galluzzi et al., 2018). Following mitochondrial depolarisation, PRKN has been proposed to inhibit pro-apoptotic BAX either by limiting recruitment of cytosolic BAX to mitochondria or by promoting degradation of dysregulated or mutated BAX (Charan et al., 2014; Cakir et al., 2017). As for BAK1, PRKN ubiquitinated a conserved residue in the hydrophobic groove of BAK1 (Lys113), thereby impairing BAK1 activation, oligomerisation and apoptotic activity. Hence, PRKN is able to inhibit BAK1 and BAX by distinct mechanisms (Bernardini et al., 2019). In this study, we proved that ailanthone upregulated BAX protein level by inhibiting BAX ubiquitination and promoted the localization of BAX in mitochondria, however, it had no significant effect on BAK1 protein expression. Therefore, we speculated that ailanthone not only triggered the release of mtDNA through the formation of BAX-BAK1 pro-apoptotic pores, thereby inducing inflammatory responses, but also potentially inhibits PRKN and activates the pro-apoptotic activities of BAX and BAK1, ultimately exerting anti-HCC effects.

**FIGURE 8:**
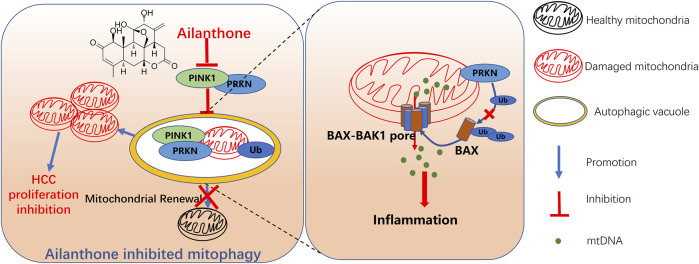
Schematic representation of ailanthone blocks mitophagy to promote mtDNA leakage through BAX-BAK1 pores and suppress hepatocellular carcinoma cell proliferation. Ailanthone induces mitochondrial damage while blocking PINK1-PRKN-mediated mitophagy, leading to the accumulation of dysfunctional mitochondria in cells, thereby inhibiting hepatocellular carcinoma (HCC) cell proliferation. Furthermore, the inhibition of PRKN protein by ailanthone reduced the ubiquitination and degradation of BAX, promoting the localization of BAX on the outer mitochondrial membrane, forming BAX-BAK1 mitochondrial pores, aggravating the release of mtDNA, and inducing inflammatory responses. Ultimately, the accumulation of damaged mitochondria and the release of inflammatory factors both contribute to the suppressive effect of ailanthone on HCC cell proliferation.

Collectively, our findings demonstrated that ailanthone from *A. altissima* exhibited anti-HCC activity and had a stronger inhibition rate in the anti-xenograft model than 5-FU *in vivo*. In addition, we preliminarily demonstrated the potential antitumour mechanism underlying ailanthone, which involved mitochondrial dysfunction, mtDNA leakage-induced inflammatory response, and inhibition of PINK1-PRKN-mediated mitophagy, ultimately leading to HCC proliferation inhibition (Figure 8). We proposed a relationship between mitochondrial homeostasis and inflammatory response and highlighted its significance in HCC treatment. Notably, this study demonstrated that ailanthone holds enormous therapeutic potential for HCC. Despite our findings, several limitations still require further investigation. It's important to determine if ailanthone poses any off-target risks, as this will help us understand its safety and specificity in clinical applications. Moreover, we need to explore the pharmacokinetics of ailanthone, which involves studying how it is absorbed, distributed, metabolized, and excreted in the body. In future studies, we will further investigate the therapeutic effects of ailanthone on HCC using PDX models and primary liver cancer models. Additionally, we plan to explore the combination of ailanthone with existing chemotherapy drugs to assess their synergistic effects and drug toxicity.

## Data Availability

The original contributions presented in the study are included in the article/Supplementary Material, further inquiries can be directed to the corresponding authors.

## Abbreviations

5-FU, 5-fluorouridine; BAK1, BCL-2 antagonist/killer 1; BAX, BCL-2-associated X; CCCP, carbonyl cyanide p-trichloromethoxyphenylhydrazone; CCK8, Cell Counting Kit 8; HE, haematoxylin-eosin; HCC, hepatocellular carcinoma; ELISA, enzyme-linked immunosorbent assay; IF, immunofluorescence; IHC, immunohistochemical; IL-1β, interleukin-1β; IL-6, interleukin-6; IP, immunoprecipitation; MitoSOX, mitochondrial reactive oxygen species; MMP, mitochondrial membrane potential; mtDNA, mitochondrial DNA; PINK1, PTEN-induced putative kinase 1; PRKN, parkin RBR E3 ubiquitin protein ligase; TEM, transmission electron microscopy; TKIs, tyrosine kinase inhibitors; TNF-α: tumour necrosis factor α.
